# 2D or 3D? How cell motility measurements are conserved across dimensions *in vitro* and translate *in vivo*

**DOI:** 10.1002/btm2.10148

**Published:** 2019-12-09

**Authors:** Sualyneth Galarza, Hyuna Kim, Naciye Atay, Shelly R. Peyton, Jennifer M. Munson

**Affiliations:** ^1^ Department of Chemical Engineering University of Massachusetts Amherst Amherst Massachusetts; ^2^ Molecular and Cellular Biology Program University of Massachusetts Amherst Amherst Massachusetts; ^3^ Department of Biomedical Engineering and Mechanics Virginia Polytechnic Institute and State University Blacksburg Virginia

**Keywords:** breast cancer, cell migration, effect size, glioblastoma, invasion, metastasis

## Abstract

Cell motility is a critical aspect of several processes, such as wound healing and immunity; however, it is dysregulated in cancer. Current limitations of imaging tools make it difficult to study cell migration *in vivo*. To overcome this, and to identify drivers from the microenvironment that regulate cell migration, bioengineers have developed 2D (two‐dimensional) and 3D (three‐dimensional) tissue model systems in which to study cell motility *in vitro*, with the aim of mimicking elements of the environments in which cells move *in vivo*. However, there has been no systematic study to explicitly relate and compare cell motility measurements between these geometries or systems. Here, we provide such analysis on our own data, as well as across data in existing literature to understand whether, and which, metrics are conserved across systems. To our surprise, only one metric of cell movement on 2D surfaces significantly and positively correlates with cell migration in 3D environments (percent migrating cells), and cell invasion in 3D has a weak, negative correlation with glioblastoma invasion *in vivo*. Finally, to compare across complex model systems, *in vivo* data, and data from different labs, we suggest that groups report an effect size, a statistical tool that is most translatable across experiments and labs, when conducting experiments that affect cellular motility.

## 1 INTRODUCTION

Cell migration is the evolutionarily conserved ability of cells to move varying distances depending on both intrinsic and extrinsic cues from their environment.^1^, ^2^, ^3^ Cell migration is vital for the development of complex, multicellular organisms during development and organogenesis.^4^, ^5^, ^6^, ^7^ Several crucial processes important to homeostasis, such as wound healing, inflammation, and angiogenesis, are dependent on cell migration.^8^, ^9^, ^10^, ^11^, ^12^, ^13^ Just as cellular motility plays a key role in normal development and function, its dysregulation has serious implications in pathobiology. Absent motility of immune cells leads to serious autoimmune diseases, chronic inflammatory conditions, and delayed wound healing.^8^, ^14^, ^15^, ^16^ Conversely, enhanced cell migration is a hallmark of cancer, with invasion of tumor cells correlating with poor patient prognosis.^17^

In order to best understand aspects of cellular motility, such as cell migration and cell invasion, we and others have developed sophisticated and controllable *in vitro* systems.^18^, ^19^, ^20^, ^21^, ^22^, ^23^ For example, synthetic biomaterials designed to mimic the extracellular matrix (ECM) allow us to conduct experiments to better understand cell movement in 3D including interactions between cells and their ECM. These *in vitro* systems, coupled with live microscopy, have allowed us to see cells move in response to extracellular signals and genetic manipulations that would be impossible *in vivo*. These analyses have been reviewed most recently by Decaesteker et al. with the merits of each system described in detail.^24^, ^25^ Importantly, the jump to 3D systems creates a more physiologically relevant environment that now requires cells to not only feel and move around on surfaces, but to also squeeze, modify, and manipulate the environment around them. *in vivo* measurements of invasion and cellular movement is difficult, though has become possible through the use of intravital imaging with fluorescently labeled cells.^26^, ^27^ However, the use of 3D *in vitro* systems is still preferred not only due to the large cost associated with using animal models, but also due to their controllability, ease of implementation, and flexibility.

There are many challenges in analyzing the data collected on cellular motility and invasion with biomaterial‐based systems. These include the diversity of assays, metrics, and analyses that result in difficulty in correlating results across platforms, stimuli, and labs. Most of the metrics used to analyze cellular invasion and motility have been developed in 2D and translated to 3D studies. We summarized the most commonly used metrics in Table 1, which include both continual live microscopy and endpoint imaging. We found cell migration reported on a population level, such as percent of cells invaded or migrating, or at a single cell level, such as migration speed or distance traveled. In this commentary, we describe the interrelation between these different motility measurements, the important differences in assays and reporting techniques used across the literature, and the potential predictive nature of *in vitro* assays to *in vivo* outcomes in a single model system.

**Table 1:** Common metrics used in the literature to determine tumor cell motility

Metric	Measurement	Description	Units	Frequency of use
% Migrating	Cell count	Number of cells moving, usually assessed by a baseline distance	%	6
% Invasion	Cell count	Number of cells that have crossed a boundary	%, fold change	10
Persistence	Fitted parameter	Time at which a cell is moving in one direction before switching	Time	4
MSD	*X*, *Y*, time	Measure of deviation of cell w.r.t. its initial position	μm^2^	1
Chemotactic index	*X* and *Y* coordinates	Net distance/ total distance	0–1	1
Net distance	*X* and *Y* coordinates	Shortest distance between the initial and final position of the cell	μm	3
Total distance	*X* and *Y* coordinates	Total distance traveled by the cell	μm	4
Speed	*X*, *Y*, time	Distance traveled by cell per unit of time	μm/min	14
Velocity	*X*, *Y*, time	Displacement of cell per unit of time (vector)	μm/min	5

## 2 RESULTS

### 2.1 Common metrics for tumor cell motility often interrelate with one another

**Figure 1:**
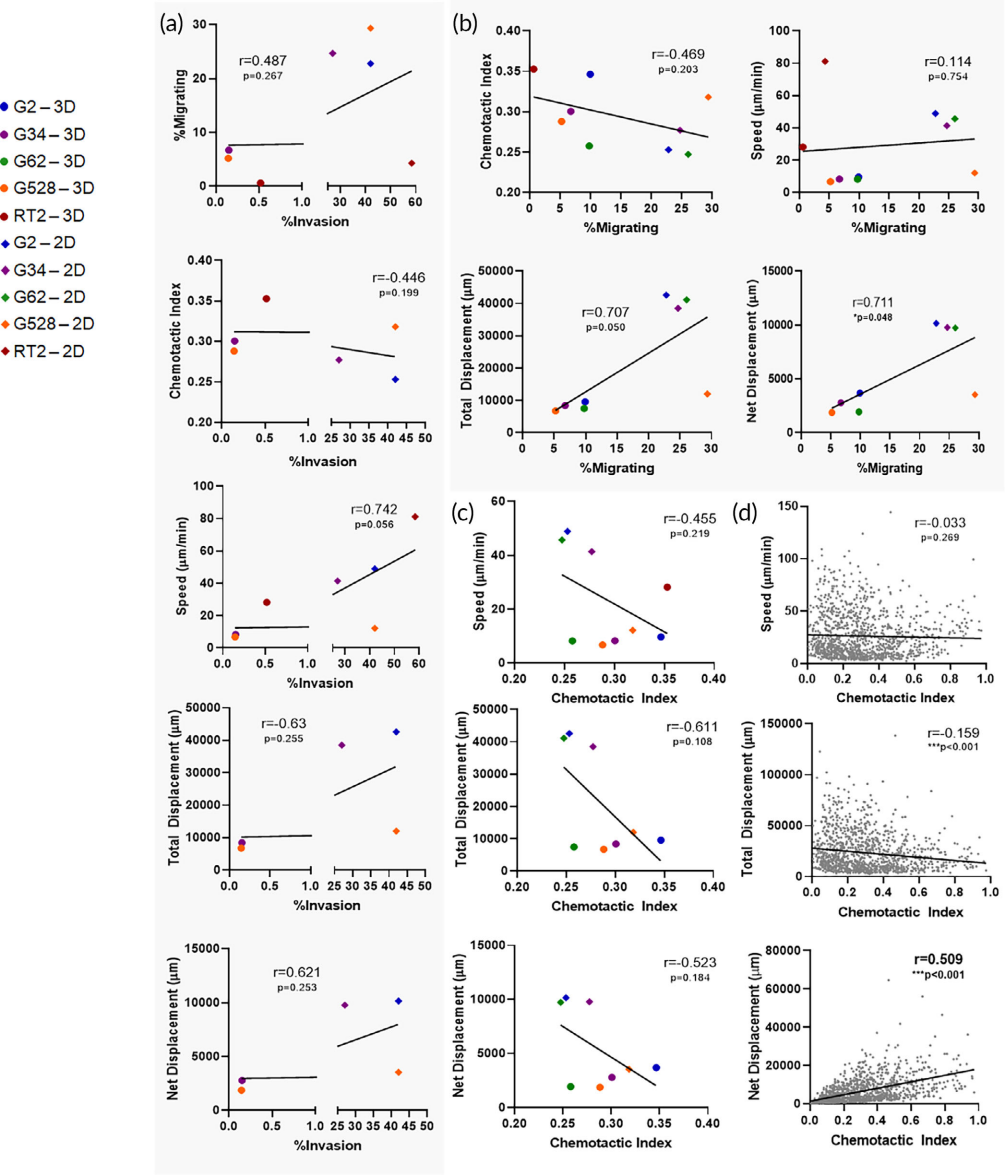
Correlations of motility outcomes for individual cell lines. (a) Metrics of motility determined by live imaging analysis and tracking versus % invasion as determined in a tissue culture insert assay. (b) Individual cell motility outcomes versus overall % of migrating cells as measured using live imaging and tracking. (c) Individual cell motility metrics averaged by cell line and dimension with (d) single cell data. Pearson *r* correlation with *p* values listed on each graph

To begin to understand how cellular motility metrics may interrelate, we analyzed the correlations between outcomes for multiple glioma cell lines by calculating the Pearson's correlation coefficient *r*, where .1 ≤ |*r*| <.3 indicates weak correlation, .3 ≤ |*r*| < .5 indicates moderate correlation, and .5 ≤ |*r*| <1 indicates strong correlation. We summarize them in Table 1, which include percent invading cells, percent migrating cells, chemotactic index, speed, total, and net displacement. Excluding percent invasion, which is a chamber‐based endpoint assay, all other metrics mentioned are obtained from live, continuous microscopy. As a first case study, we compared live imaging and percent invasion data for several patient‐derived glioma stem cell (GSC) lines, including G2, G34, G62, and G528 (Figure 1, Figure S1). We first compared motility metrics assessed with live imaging to endpoint percent invasion and determined that no single metric significantly correlated with this endpoint metric (Figure 1a, *p* > .05). Although they are not statistically significant, there was a moderate correlation (.3 ≤ |*r*| <.5) for chemotactic index (*r* = −.446, *p* = .199) and a strong correlation (0.5 ≤ |*r*| ≤1) for the speed (*r* = .742, *p* = .056). Next, we aimed to determine if there was a correlation between the percent of migrating cells in a total population and single cell metrics of motility (Figure 1b) and identified that both total and net displacement positively correlated with the total percent of cells that were migrating (*r* = .707 and .711, respectively, *p* < .05). Finally, we compared the single cell metrics of motility based on tracts of individual cells to identify correlations both averaged for the total population (Figure 1c) and of the single cells (Figure 1d, *n* = 1,182 cells tracked). We found an expected positive correlation between net displacement and speed (Figure S1a, *r* > .98, *p* < .001), and between displacement and chemotactic index for both the population averaged outcomes (Figure 1c) and the individual cell measurements (Figure 1d). The correlations with percent invasion are particularly interesting as the invasion of cells *in vitro* is often assumed to be predictive of invasiveness *in vivo*. Overall, these correlations indicate that it may be possible to infer some cellular motility behaviors from a single assay/measurement. This may be important when making decisions regarding experimental design and analysis of data.

### 2.2 For glioblastoma cell lines, 2D motility correlates with 3D motility

**Figure 2:**
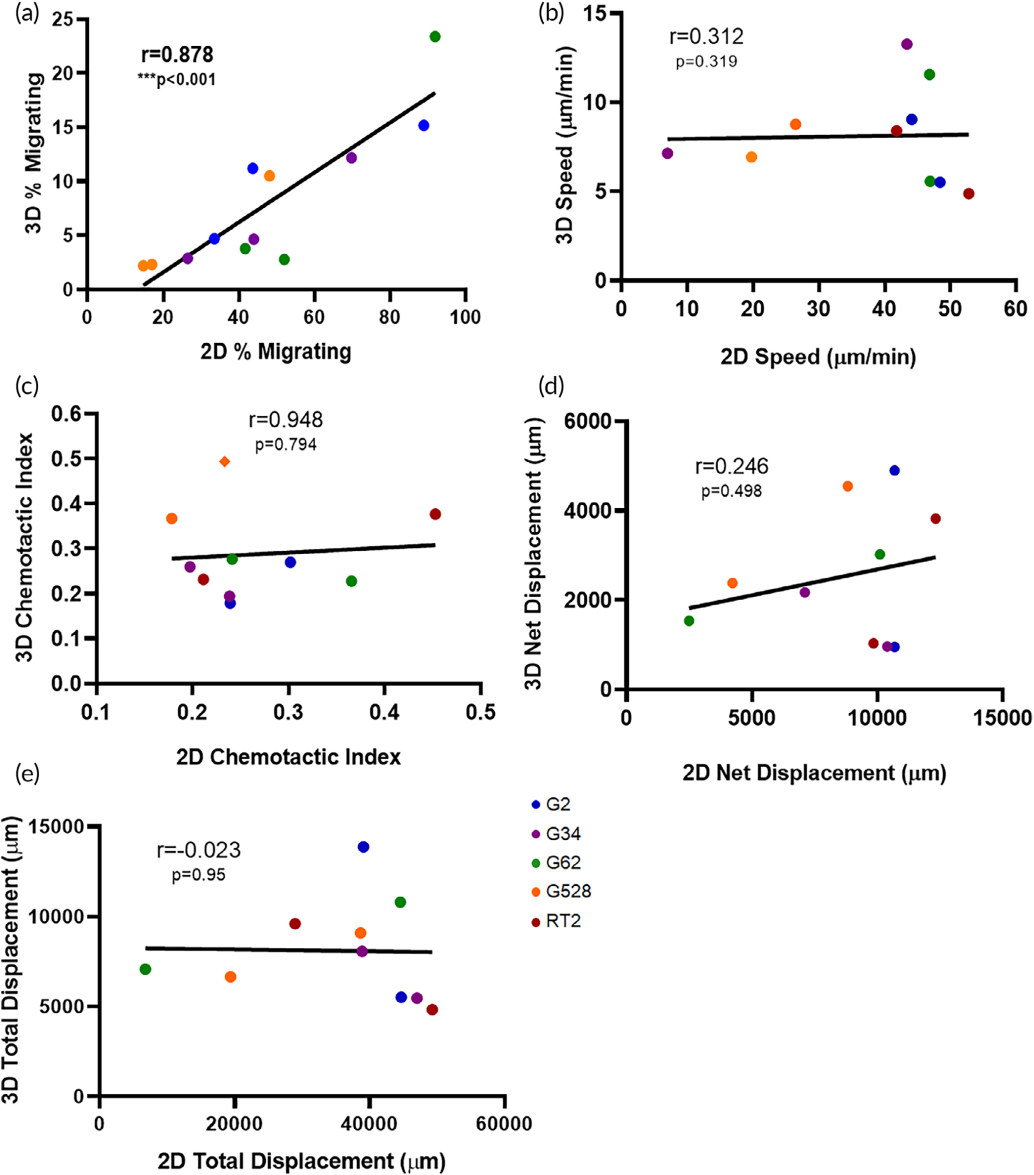
Motility metrics compared in 2D and 3D environments for glioma cells. Averaged motility outcomes determined from live imaging and tracking are shown for individual experimental runs and correlated by glioma cell line. (a) Percent of cells migrating greater than two cell lengths. (b) Speed of cells, (c) chemotactic index, (d) net displacement, and (e) total displacement as determined from individual tracks. Pearson *r* correlation with *p* values listed on each graph

Although cellular motility in 2D and 3D microenvironments entail many of the same underlying mechanisms of cellular motion including contractility, adhesion, and cytoskeletal rearrangement, 3D systems are thought to better mimic *in vivo* conditions by surrounding cells with the ECM. Given the increased use of 3D environments in which to study cells, we sought to evaluate what measurements of 2D motility might translate to cell migration in 3D. Using glioma as a case study, we compared the 2D and 3D motility measurements (Figure 2) across experiments with four GSC lines and one glioma cell line by calculating correlation coefficients (Pearson's *r*) and *p* values. Comparing percent migrating cells, speed, net distance, and chemotactic index in 2D versus 3D environments showed that only one metric—percent of migrating cells—correlated significantly between 2D and 3D (Figure 2a, *r* = .878, *p* < .001). Generally, the total percentage of cells migrating was significantly higher in 2D than in 3D, as explained by a linear regression ([2D] = 3.3 × [3D] + 21.2). Speed of cells migrating was also lower in 3D than in 2D, as has been commonly reported.^28^, ^29^, ^30^, ^31^, ^32^ Observationally, the range of chemotactic indices was strongly correlated, though not statistically significant, between 2D and 3D (Figure 2c, *r* = .948). When comparing the total and net displacement in 3D compared to 2D culture, there were weak correlations in between as well as statistically not significant. Thus, we were surprised to see that many metrics of individual cell motility did not correlate between 2D and 3D, though the total percent of migrating cells did.

### 2.3 No obvious relationship between measurement time or cell density and cell migration quantification from the literature

**Figure 3:**
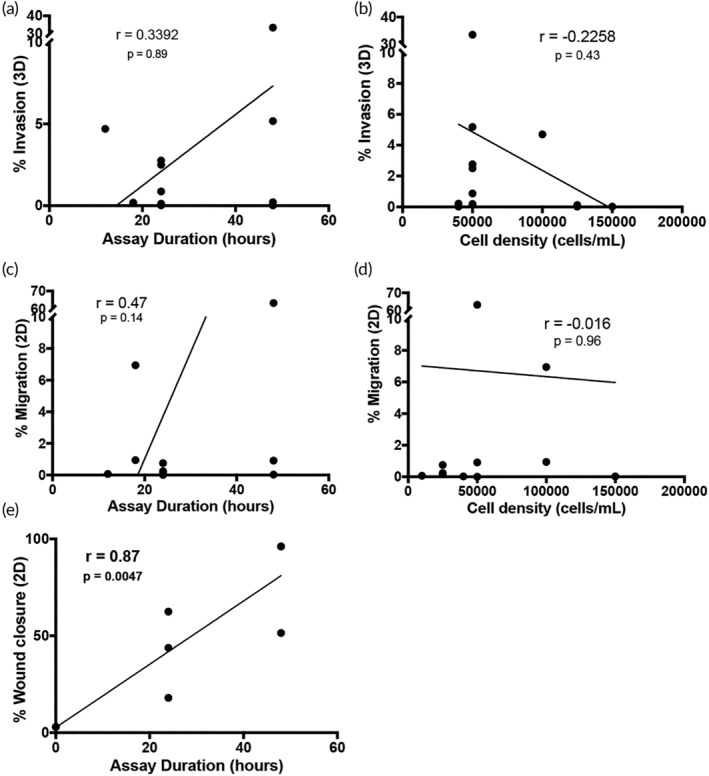
Correlation of experimental set up and outcomes from literature for tumor cells. Compiled data outcomes from existing experiments in the literature that examine tumor cell motility as compared to assay parameters. (a) Percent invasion in a tissue culture insert‐Matrigel assay versus duration of the experiment and (b) initial cell seeding density. (c) Percent of cells migrating through tissue culture inserts (without Matrigel) versus the duration of the experiment and (d) initial cell seeding density. (e) Percent of wound closure in traditional 2D scratch assay versus the duration of the experiment. Pearson *r* correlation with *p* values listed on each graph

The data in Figures 1 and 2 are a result of experiments performed in a single lab, and thus, potential confounding factors such as the culture medium, culture substrate, type and length of assay, and interpretation of data were largely controlled for. However, across the literature, cellular motility is examined not only via different metrics and assays, but also with varying experimental setup. Thus, we aimed to examine the variability in assay set up and its potential effects on outcomes through a careful literature search focused on several of the most widely examined cell lines in motility assays. We compiled data from a list of publications measuring motility in 2D and 3D platforms (Figure 3 and Tables S1–S6) among widely used cell lines to extrapolate our findings to that beyond our own labs. We focused on studies of cell motility in 3D that reported % invasion (Figure 3a,b) and % migrating (Figure 3c,d), and studies that reported % wound closure in 2D (Figure 3e). We saw no significant correlation for the 3D motility outcomes with the two consistent experimental conditions reported (assay duration and cell density). In the case of wound healing assays, however, there was an unsurprising correlation between assay duration and percent of wound closure (*r* = .87, *p* < .01) (Figure 3c).

We found that biomaterial properties like pore size and composition were similar across studies, although concentrations of basement membrane extract (*i.e*., Matrigel®) used were often not reported (Tables S1–S2). Cell invasion outcomes from tissue culture insert assays were reported differently across publications and included total cell number, self‐defined “invasion value,” fold change, percent invasion, or images without quantitative metrics (Table S3). Assay readouts varied significantly between crystal violet, H&E staining, trypsinization prior to counting, or simply imaging counting, all at different time points (Tables S3–S5). In the case of invasion, attractants used in invasion assays were unique to each study (Table S6). Thus, we could not determine a correlation between the assay experimental setup and the cell migration‐related outcomes. We were also unable to quantitatively evaluate all experimental design components (such as matrix concentration) within this small sample size of publications. Similarly, when examining live imaging data in Collagen I matrices, another popular substrate for tumor cell motility assays, we saw a high degree of variability in metrics measured across 10 studies including percent migrating and cell speed (Figure S4).

### 2.4 In vivo invasion in glioma negatively correlates with 3D chemotactic index

**Figure 4:**
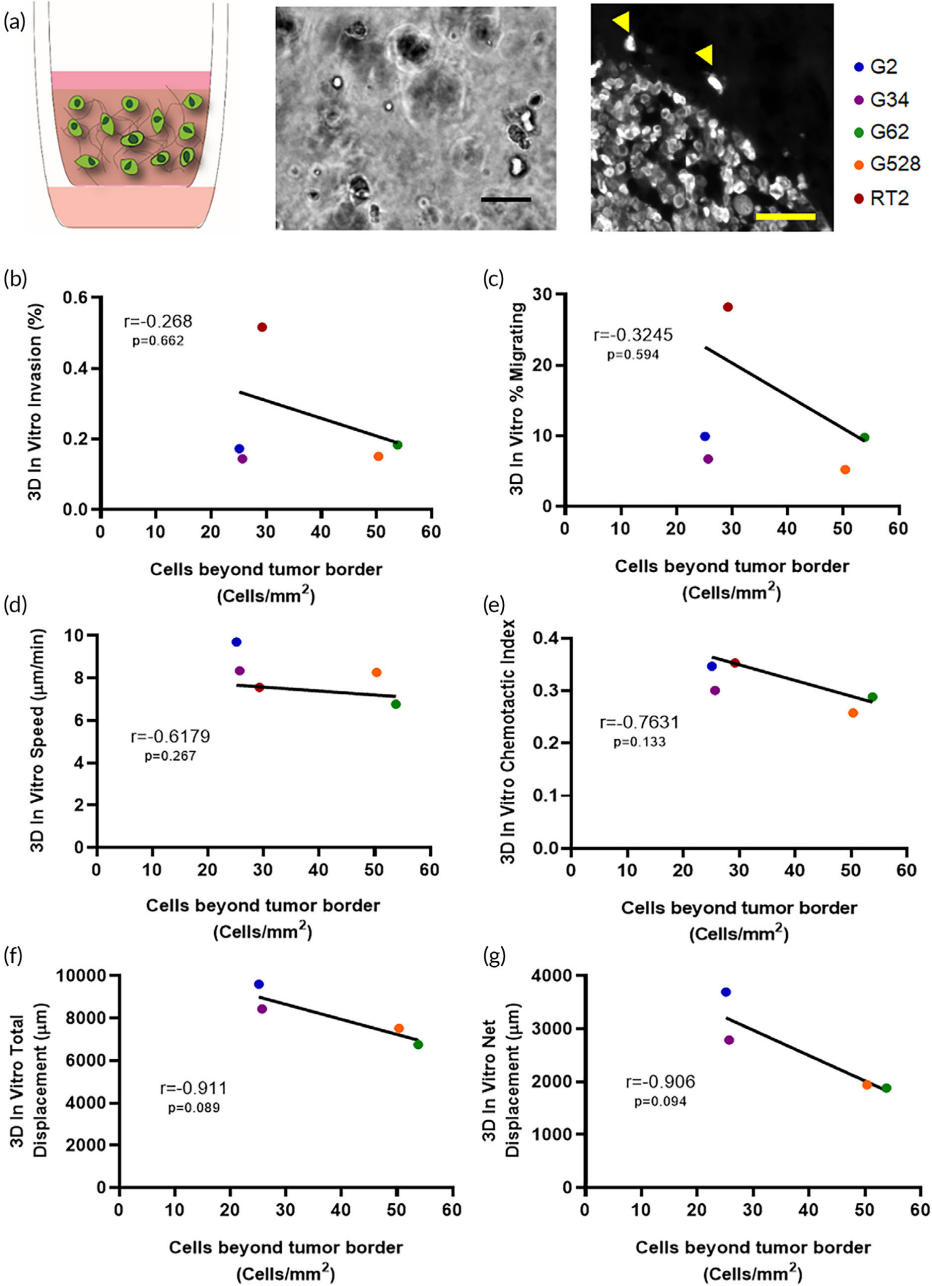
Motility metrics compared in a 3D environment *in vitro* to *in vivo*. **(**a) From left to right the images represent the* in vitro* invasion assay, live imaging micrograph from cells in a 3D hyaluronan matrix *in vitro* and glioma cells implanted in mouse brain at the tumor border with invasive cells beyond the border (arrowheads). **(**b) *in vitro* percent invasion, (c) percent cells migrating, (d) speed, (e) chemotactic index, (f) total displacement, and g) net displacement graphed by glioma cell line versus the number of invaded cells beyond the tumor border in vivo per mm^2^ of tissue. Pearson *r* correlation with *p* values listed on each graph

One major stated goal of *in vitro* assays is to predict, or at least model, cell movement in order to better understand the mechanistic underpinnings and driving factor of cell movement *in vivo*. For glioblastoma (GBM), the deadliest form of brain cancer, invasion is a hallmark of its behavior and is responsible for recurrence after treatment. Unlike other cancers, in GBM, invasive cells remain within the primary organ, which allows for straightforward quantification of invasion at an endpoint using immunohistochemistry. We hypothesized that this invasion would positively correlate with outcomes of cellular motility *in vitro*. Using previously published data from five models of GBM (our four GSC lines and the rat glioma line RT2) implanted into rodent cortex, we quantified cells that had invaded beyond the tumor border and correlated these numbers to our assays *in vitro* (Figure 4a). Results from at least four mice were averaged (data from Reference 33) and plotted against averaged values from at least four *in vitro* experiments. For cells in 3D, we did not see a statistically significant correlation between any motility metric *in vitro* and our *in vivo* results (Figure 4b–g). However, we did see a moderate negative correlation for 3D chemotactic index (Figure 4e) and strong negative correlations for both net and total displacement (Figure 4g) with *in vivo* invasion. In 2D, we saw a strong positive, though not significant, correlation only when comparing percent migrating cells (Figure S2a) with the invasion metric* in vivo*. Due to our low number of cell lines to compare in vitro and in vivo, it is difficult to draw concrete conclusions about invasion *in vitro* and *in vivo*, though we see interesting negative trends that are contrary to our current assumptions about translating *in vitro* invasion outcomes to *in vivo* results. These data were generated from the same lab using a single biomaterial system and can thus be analyzed together, but an ability to examine data across labs, tumor models, and *in vitro* models would allow us to better interpret *in vitro* and *in vivo* correlations. For this, unified metrics are necessary so that we can easily compare between studies within and between laboratories.

### 2.5 Effect size as a statistical tool to measure motility changes across dimensions

**Figure 5:**
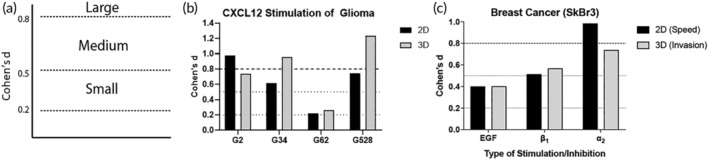
Motility effect sizes for tumor cells in 2D or 3D. (a) Cohen's *d* (effect size) delineations for small (≥0.2, <0.5), medium (≥0.5, <0.8), and large (≥0.8) effect sizes. (b) Cohen's *d* calculated for percent migrating cells when stimulated with CXCL12 versus vehicle control of patient‐derived glioma stem cell lines in 2D and 3D. (c) Cohen's *d* for SkBr3 breast cancer cells when stimulated with epidermal growth factor or treated with inhibitors of integrin‐*β*_1_ or integrin‐*α*_2_ in 2D (for speed of cell migration) or 3D (for invasion into collagen gels)

Mechanistic invasion and motility assays aim to determine the response to particular stimuli or inhibitor (and determine if that difference is statistically significant from some internal control). It is often assumed, though not directly tested, that if a stimulus increases 2D motility it will do the same in 3D. To test this assumption, we revisited our previous data and calculated effect sizes (Cohen's *d*) in 2D and 3D to determine if (a) dimensionality alters the effect of stimuli and (b) we can use effect size to better analyze and compare cell motility in response to stimuli across dimensions. Unlike the *r* and *p* values we have used above to compare correlations between two different cell motility metrics, here we used effect sizes to quantify and compare the size of the difference between two groups. Effect size is a statistical concept that defines the strength of a relationship between two variables or conditions on the same numeric scale.^34^ Effect size uses Cohen's *d* value as an indicator, with Cohen's *d* defined as the difference between two means divided by the *SD*. Cohen et al. states that when the Cohen's *d* is lower than 0.2, there is no effect. If the value is 0.2 ≤ |*d*| <0.5, there is a “small” effect, a “medium” effect if the value is 0.5 ≤ |*d*| <0.8, and a “large” effect when |*d*| ≥0.8 (Figure 5a). Thus, using this value, one can easily compare the effect of one treatment to another regardless of laboratory, experimental setup, or outcome measure to determine how universal findings are.

#### 2.5.1 Glioma motility in response to CXCL12

We examined motility of multiple patient‐derived GSC lines in the presence of 100 nM of CXCL12 in 2D and 3D (Figure 5b) by reanalyzing our previously published data.^33^ CXCL12 is a pro‐migratory chemokine that has been implicated in glioma motility and invasion.^35^ We quantified multiple outcomes with live cell tracking and found that the effect size varied based on the dimensionality. For some cell lines (G62) the effect size was nearly equal for percent motile cells when cells were stimulated in 2D or 3D and indicated that there was a small effect (<0.2) of the stimulation. For G2 and G528, the effect size varied but remained large (≥0.8) for both cell lines in both dimensions. Interestingly though, for G34, the effect in 2D was medium, but large in 3D, indicating that dimensionality may affect this cell line‐specific response to CXCL12.

#### 2.5.2 Breast cancer motility in response to EGF and integrin inhibitors

To broaden the utility of effect size beyond glioma to breast cancer cell behavior, Figure 5c shows SkBr3 cells that were seeded on a bone‐ECM functionalized surface and stimulated with epidermal growth factor (EGF) or inhibitors for integrin subunits *β*_1_ and *α*_2_.^36^ EGF stimulation ultimately leads to cell proliferation, and integrins are necessary for cell‐ECM binding, cell migration, and invasion. The original study used Spearman correlation and *p* values to validate correlations among different cell motility metrics^36^; however, it did not allow us to compare the effect of each stimuli or inhibition on 2D versus in 3D. EGF stimulation had a small effect, and β_1_ integrin inhibition had a medium effect, in both 2D and 3D. In addition, *α*_2_ integrin inhibition had a large effect on 2D, but a medium effect in 3D. Our analysis highlights the utility of using the statistical tool effect size to determine its importance given its ability to span dimensionality and cell sources.

## 3 DISCUSSION

In this analysis, we found that the diversity of invasion and motility assay measurement approaches, reporting tools, and responses all vary across labs (Figure 3 and Tables S1–S6). Although motility metrics have been studied in multiple contexts for decades, there is still not a consensus nor clarity in terms of the importance of each and the impact of each on outcomes *in vivo*. In cancer, this is particularly striking, as there is already a high level of heterogeneity in the disease itself, which is amplified as we move into complex *in vitro* models. One major impediment to the field's progress is the variability from lab to lab in the implementation and analysis of these experiments. First, we identified high variability in the assay setup. As illustrated in Table S1, concentrations of Matrigel® used for invasion assays differed, and in some publications, were not reported. We know that the source and the lot of basement membrane extracts (like Matrigel®) can influence experiments alone, let alone the concentration.^37^ Similarly, assay durations and cell densities differed across most publications using breast cancer cell lines (Tables S3–S5). Unsurprisingly, the assay duration correlated positively with degree of wound closure (Figure 3e). When we looked through how different publications quantified their assay outcomes, we noticed variable methods to count invasive cells from the bottoms of tissue culture inserts, including selection of immunocytological stain and/or fixation versus cellular detachment and counting. Regardless, publications generally reported some final number, though this could be a percent, fold change, or total number of cells that prevented us from directly comparing their results as were able to do for our own experiments. A standardized metric that best conveys the raw data would allow to compare outcomes in a meaningful way across labs.

We propose effect size as a useful metric to understand how and if stimuli and inhibitors affect cell motility across geometries and labs. For example, as seen on Figure 5b,c, comparing each Cohen's *d* value illustrates the effect of each ECM substrate or each stimulus for two different cell types. Within each cell line, we can see the significant effect of the stimulus on cell response, across geometries, and independent of the cell's genetic background. Additionally, comparing the value of the effect size (≥0.2, ≥0.5, or ≥ 0.8) allows us to better understand how large an effect is, without the need for a *p* value (which has been recently put into question^38^). Not only does it allow us to characterize two effect sizes in the same category, but it also gives us a better understanding on whether there are large differences or not. For example, if the effect size is <0.2, it means the two comparing group's means do not differ by 0.2 *SD*s or more, which indicates the difference is small even if it might be statistically significant. In this way, the effect size allows us to better quantify the real effect of a stimulus on an experimental group compared to control, independent of a *p* value.

The desire to understand how 2D cell migration relates to that in 3D is not unique to our study. Meyer et al. quantified breast cancer cell line motility and showed that the degree of initial cell protrusion in 2D was predictive of 3D invasion across many different stimuli.^29^ In agreement with or analysis of glioma cells, Meyer et al. found no other obvious correlations between 2D and 3D cell migration measurements. Similarly, when studying the role of focal adhesion proteins in cellular motility, Fraley et al. compared speed, persistence, protrusion length/number/time, etc. in 2D and 3D and found no correlation between any of the metrics in the two environments.^39^ Next generation biomaterials are being developed that provide possible explanations of the key differences between 3D and 2D environments that drive the unique motility phenotypes, such as confinement^40^, ^41^ and porosity.^28^

Many labs are quantifying cell invasion *in vivo* in order to potentially discover druggable targets to halt malignant cells from invading and metastasizing. 3D microenvironments have been lauded as “more physiologically relevant,” but in our limited data set we show that there is no significant correlation (slight negative trend) between most motility metrics in 3D collagen/hyaluronan gels and invasion *in vivo*. Live imaging data *in vivo* may reveal more information, but with at least this endpoint assay, we cannot predict *in vivo* “invasiveness” with *in vitro* invasion in glioma. This result is not altogether unsurprising in that the movement between dimensions and into a more complex system includes many changes to biophysical interactions. Thus, it is possible that our *in vitro* systems, even in 3D, do not have enough complexity to capture true *in vivo* behavior, such as additional cell‐to‐cell interactions, growth factors, cytokines, and specific integrin binding sites to the ECM. Further, it may be that we may never fully predict specific behaviors that translate in vivo, yet the information that we gain is still valuable for fundamental understanding of cell motility.

Taken together, standardized metrics are needed that allow for direct comparison between 2D, 3D, and in vivo models. Effect size can allow us to better compare the effects of different stimuli on motility metrics and perhaps draw conclusions independent of dimension and environment. Given the rise of more physiological *in vitro* models that result in more complicated responses, this could be a first step to implement comparison of metrics across the field. Finally, standardizing motility metric outcomes could help bridge the gap between 2D, 3D *in vitro* systems, and their translation to *in vivo* physiology.

## 4 MATERIALS AND METHODS

### 4.1 Cell culture

All cell culture supplies were purchased from Thermo Fisher Scientific (Waltham, MA) unless otherwise noted. The SkBr3 cell line was purchased from ATCC (Manassas, VA), and cells were grown in DMEM, supplemented with 10% fetal bovine serum (FBS) and 1% penicillin/streptomycin (pen/strep).

### 4.2 Preparation of ECMs for SkBr3 migration experiments

Glass coverslips (15 mm and 18 mm diameter, Fisher Scientific, Agawam, MA) were functionalized with 10 g/L *N*,*N*‐disuccinimidyl carbonate (Sigma‐Aldrich) and 5% vol/vol diisopropylethylamine (Sigma‐Aldrich), and ECM protein cocktails were then covalently bound to the glass coverslips through reactive amines: 5 μg/cm^2^ of 99% Collagen I and 1% osteopontin.^36^ Coverslips were incubated with proteins at room temperature for 3 hr, rinsed three times with PBS, and then incubated with 10 μg/cm^2^ MA(PEG)24 (Thermo Scientific, Rockford, IL) for 2 hr. Coverslips were rinsed three times with PBS, epoxied to the plate (Devcon 5 min epoxy) and UV‐sterilized prior to cell seeding. For invasion studies from coverslips, cells were seeded on coverslips and then overlaid with a collagen gel as previous described.^36^

### 4.3 3D invasion assays analysis

Invasion assay data for glioma cells was acquired from our previous publications where it was conducted as described.^33^, ^42^ Membranes were imaged at five non‐overlapping locations and % invasion was calculated as an extrapolated cell count divided by the seeded cell count × 100. Data included in this publication were taken from our previous publications for RT2, G2, G34, G62, and G528.^33^, ^42^

### 4.4 Live imaging analysis

#### 4.4.1 Glioma motility

The motility metrics were determined via live imaging and single‐cell tracking of glioma cells from previously acquired and published images. Images taken in 20‐min intervals for 18–24 hr were analyzed for cell motility metrics. The manual tracking feature on Celleste 4.1 was used to record the location of the visually identified center of the cell of interest in each image of the sequence. An average of 15 cells was tracked per image. The recorded *X* and *Y* coordinates were analyzed in MATLAB 2018b with the following outcomes: average speed, net and total displacements, and chemotactic index of each cell. Two to nine image sequences were analyzed per cell type (G528, G62, G34, and G2^33^) and experimental condition (2D, 3D) combination per experiment. The averaged values per experiment are reported here. Data for RT2 were taken from previous publication.^42^

#### 4.4.2 SkBr3 motility

Cells were seeded at 4,000 cells/cm^2^ on ECM protein treated surfaces. They were then treated with a live‐cell fluorescent dye (CMFDA, Life Technologies), and fresh medium or medium supplemented with EGF and/or integrin antibodies were provided 4 hr prior to microscopy. Brightfield and fluorescent images were taken at 15‐min intervals for 12 hr using an EC Plan‐Neofluar 10× 0.3 NA air objective (Carl Zeiss). Cells were tracked using Imaris (Bitplane, St. Paul, MN) to generate individual cell paths, and individual cell speeds were determined by calculating a speed at every 15‐min time interval, then averaging these over the entire 12 hr.

### 4.5 Tumor inoculation

Tumor images from previous publications were reanalyzed to determine the number of cells migrated per area beyond the tumor border. Original experiments were approved by Institutional Animal Care and Use Committees as described in those publications. After importing raw images into ImageJ, cells were counted in four to five 0.49 mm^2^ regions of the image. RT2 glioma cell line in rat^43^; G2, G34, G528 GSCs in SCID mice^33^; and G62 GSC in SCID mice.^44^

### 4.6 Invasion calculations from published data

Percent of invasion, and migration data were extracted with the WebPlotDigitizer v4.1 from the published work cited in Figure 3 and Tables S1–S6. Re‐plotted data were used to calculate the percent of invasion based on the initial number of seeded cells.

### 4.7 Effect size calculations

Effect size measures were performed between two independent groups following Cohen's *d* calculation:

$$d=M_{1}-M_{2}/s_{\text{pooled}}$$

$$s_{\text{pooled}}=\sqrt{\left(s_{1}^{2}+s_{2}^{2}\right)/2}$$

Here, *M*_1_ and *M*_2_ are the means of two independent samples being compared (e.g., control vs. experimental group), and *s*_pooled_ is the pooled *SD* where *s*_1_ and *s*_2_ are the *SD*s of the Groups 1 and 2, respectively. We used the online calculator from Dr. Lee A. Becker at the University of Colorado, Colorado Springs at https://www.uccs.edu/lbecker/.

## 5 CONCLUSION

Current challenges in the field of cellular motility and invasion within biomaterial‐based systems, including diversity of assays, metrics, and analyses, limit the translation of results across platforms and impede correlation between 2D, 3D and *in vivo*. Here, we summarize the most commonly used metrics to quantify cell motility, and describe the interrelation between these different motility measurements, the important differences in assays and reporting techniques used across the literature, and describe the potential contribution of *in vitro* predictions to *in vivo* outcomes. To our surprise, we found cell invasion in 3D has a weak negative correlation with invasion in a glioblastoma model *in vivo*. Given the variability we saw in reporting in the literature, and the inability to predict 3D or *in vivo* invasion from simpler 2D assays, we suggest that standardized metrics are needed. We recommend the use of effect size as a possible avenue that allows direct comparison between two different groups independent on dimensionality or stimulus. Given the rise of more physiological *in vitro* models that result in more complicated responses, this could be a first step to implement comparison of metrics across the field. Finally, standardizing motility metric outcomes could help bridge the gap between 2D, 3D *in vitro* systems and their translation to *in vivo* physiology.

## Supporting information


**Figure S1: Correlation of metrics related to cellular displacement:** A) Comparing total displacement, net displacement, and speed for the following four cell lines in 2D and 3D environments: G2, G34, G62, G528. B) Single cell data comparing the metrics mentioned previously for all cell lines and dimensions.
**Figure S2: Motility metrics for glioma cells in 2D *in vitro* compared to *in vivo* invasion.** a) Percent of cells migrating, b) chemotactic index, c) speed, d) total displacement, and e) net displacement graphed by glioma cell line vs. the number of invaded cells beyond the tumor border in vivo per mm^2^ of tissue. Pearson *r* correlation with *p* values listed on each graph.
**Figure S3. Invasion of MDA‐MB‐231 cell line correlates with metastasis.** I*n vivo* effect sizes compared to tissue culture polystyrene (TCPS) for cells cultured in soft (1 kPa) or stiff (41 kPa) hydrogels prior to implantation.
**Table S1:** Concentration of basement membrane extract (i.e., Matrigel) used in tissue culture insert invasion assay experiments
**Table S2:** Tissue culture inserts used in assays with tumor cells
**Table S3:** Cell seeding and invasion metric data for tissue culture insert tumor cell invasion assays from the literature
**Table S4:** Assay readout for tissue culture insert invasion assays
**Table S5:** Tissue culture insert migration assay readout
**Table S6:** Type of medium used in tissue culture insert invasion assays in lower chamber
**Figure S4: Motility metrics for MDAMB231 cultured in Collagen I matrices and live imaged** A) Cell speed measured in 3D across studies B) % of cells migrating in 3D by study C) Table of studies from which data was extracted.
