# Supplementary material for: 2D or 3D? How cell motility measurements are conserved across dimensions in vitro and translate in vivo
Source: Bioeng Transl Med. 2019 Dec 9;5(1):e10148. doi: 10.1002/btm2.10148 (PMC6971446; doi:10.1002/btm2.10148)
Supplement: Supplementary file 1 — Figure S1: Correlation of metrics related to cellular displacement: A) Comparing total displacement, net displacement, and speed for the following four cell lines in 2D and 3D environments: G2, G34, G62, G528. B) Single cell data comparing the metrics mentioned previously for all cell lines and dimensions. Figure S2: Motility metrics for glioma cells in 2D in vitro compared to in vivo invasion. a) Percent of cells migrating, b) chemotactic index, c) speed, d) total displacement, and e) net displacement graphed by glioma cell line vs. the number of invaded cells beyond the tumor border in vivo per mm2 of tissue. Pearson r correlation with p values listed on each graph. Figure S3. Invasion of MDA‐MB‐231 cell line correlates with metastasis. In vivo effect sizes compared to tissue culture polystyrene (TCPS) for cells cultured in soft (1 kPa) or stiff (41 kPa) hydrogels prior to implantation. Table S1: Concentration of basement membrane extract (i.e., Matrigel) used in tissue culture insert invasion assay experiments Table S2: Tissue culture inserts used in assays with tumor cells Table S3: Cell seeding and invasion metric data for tissue culture insert tumor cell invasion assays from the literature Table S4: Assay readout for tissue culture insert invasion assays Table S5: Tissue culture insert migration assay readout Table S6: Type of medium used in tissue culture insert invasion assays in lower chamber Figure S4: Motility metrics for MDAMB231 cultured in Collagen I matrices and live imaged A) Cell speed measured in 3D across studies B) % of cells migrating in 3D by study C) Table of studies from which data was extracted. [file BTM2-5-e10148-s001.docx]

Supplemental Table 1: Concentration of basement membrane extract (i.e. Matrigel) used in tissue culture insert invasion assay experiments

| Cell line | BME Concentration | Reference |
| --- | --- | --- |
| MDA-MB-231 | not specified | ^41^ |
| MDA-MB-231 |  | ^42^ |
| MDA-MB-231 |  |  |
| MCF7 | 50 µg/ml | ^43^ |
| SKBR3 | not specified | ^44^ |
| SUM149 |  | ^41^ |
| HMECs |  |  |
| PC3 | 50 µg/ml | ^45^ |
| DU-145 |  |  |
| MDA-MB-231 | 0.2umol | ^46^ |
| T-47D |  |  |
| Hs578T | not specified | ^47^ |
| MCF10A |  |  |
| BT549 |  | ^42^ |

Supplemental Table 2: Tissue culture inserts used in assays with tumor cells

| Cell line | Pore size (µm) | Material of membrane | Reference |
| --- | --- | --- | --- |
| MDA-MB-231 | 8 | polycarbonate | ^41^ |
| MDA-MB-231 | 8 |  | ^42^ |
| MDA-MB-231 | 8 |  |  |
| MCF7 | 0.8 | polyethylene terephthalate | ^48^ |
| SKBR3 | ND | ND | ^44^ |
| SUM149 | 8 | polycarbonate | ^41^ |
| HMECs | 8 |  |  |
| PC3 | 8 |  | ^45^ |
| DU-145 | 8 |  |  |
| MDA-MB-231 | 8 | polyethylene terephthalate | ^46^ |
| T-47D | 8 |  |  |
| Hs578T | 8 | polycarbonate | ^47^ |
| MCF10A | 8 |  |  |
| BT549 | 8 |  | ^42^ |

Supplemental Table 3: Cell seeding and invasion metric data for tissue culture insert tumor cell invasion assays from the literature

| Cell line | Initial cells seeded | Invasion Data Format | Reported Invasion Value | Reference |
| --- | --- | --- | --- | --- |
| MDA-MB-231 | 125,000 cells | Cell Per Field | 147.8 cell per field | ^41^ |
| MDA-MB-231 | 250,000 cells | Invasion Value | 89 invasion value | ^49^ |
| MDA-MB-231 | 50,000 cells/well | Number of invaded cells | 1381.49 invaded cells | ^42^ |
| MDA-MB-231 | 50,000 cells/well |  | 434.78 invaded cells | ^42^ |
| MDA-MB-231 | 200,000/ml cells | Fold Change | 2.45 fold | ^48^ |
| MCF7 | 100,000 cells | Percentage Invasive Cells | 4.70% | ^43^ |
| SKBR3 | 150,000 cells | Number of invaded cells | 30.28 of invasive cells | ^44^ |
| MDA-MB-435 | 200,000/ml cells | Fold Change | 1.95 fold | ^48^ |
| SUM149 | 125,000 cells | Cell Per Field | 20 cell per field | ^41^ |
| HMECs | 125,000 cells |  | 52.44 cell per field | ^41^ |
| PC3 | 50000 cells |  | 96.3 cell per field | ^45^ |
| DU-145 | 50000 cells |  | 81.91 cell per field | ^45^ |
| MCF7 | 100000 cells | image only cannot quantify |  | ^50^ |
| MDA-MB-435 | 100000 cells | image only cannot quantify |  | ^50^ |
| MDA-MB-231 | 40,000 cells | cell per field | 85.1 transmembrane cells | ^46^ |
| T-47D | 40,000 cells | cell per field | 8.97 transmembrane cells | ^46^ |
| Hs578T | 50,000 cells | cell number | 16744 cell number | ^47^ |
| MCF10A | 50,000 cells | cell number | 2590 cell number | ^47^ |
| BT549 | 50,000 cells/well | average cell per field | 1249 average cell # per field | ^42^ |

Supplemental Table 4. Assay readout for Tissue Culture insert invasion assays

| Cell line | Endpoint (hours) | Reference |
| --- | --- | --- |
| MDA-MB-231 | 24 | ^41^ |
| MDA-MB-231 | 24 | ^42^ |
| MDA-MB-231 | 24 | ^42^ |
| MCF7 | 12 | ^43^ |
| SKBR3 | 48 | ^44^ |
| SUM149 | 24 | ^41^ |
| HMECs | 24 | ^41^ |
| PC3 | 18 | ^45^ |
| DU-145 | 18 | ^45^ |
| MDA-MB-231 | 48 | ^46^ |
| T-47D | 48 | ^46^ |
| Hs578T | 48 | ^47^ |
| MCF10A | 48 | ^47^ |
| BT549 | 24 | ^42^ |

Supplemental Table 5. Tissue Culture Insert migration assay readout

| Cell line | Experiment time | Analysis method for readout | Reference |
| --- | --- | --- | --- |
| MCF7 | 18 h | staining with 0.5% crystal violet avg. migrated cells bound per field | ^50^ |
| MCF7 | 0h, 12h, 36h | stained and counted | ^43^ |
| MDA-MB-231 | 24 h | stained with Diff-Quick staining set and counted | ^41^ |
| MDA-MB-231 | 48 hours | fixed 4% PFA, stained hematoxylin and eosin, counted | ^46^ |
| MDA-MB-231 | 24-48hrs | fixed 10% formalin, stained 0.05% crystal violet. Distance migration from spheroid center | ^42^ |
| MDA-MB-231 | 24 hours | fixed methanol, stained crytal violet. | ^48^ |
| MDA-MB-435 | 18 h | staining with 0.5% crystal violet avg. migrated cells bound per field | ^50^ |
| MDA-MB-435 | 24 hours | fixed methanol, stained crytal violet. | ^48^ |
| MDA-MB-453 |  | stained and counted | ^43^ |
| SKBR3 | 24 hours | fixed methanol, stained 4 g/L crystal violet | ^44^ |
| BT549 | 24-48hrs | fixed 10% formalin, stained 0.05% crystal violet. Distance migration from spheroid center | ^42^ |
| SUM149 | 24 h | stained with Diff-Quick staining set and counted | ^41^ |
| HMECs | 24 h | stained with Diff-Quick staining set and counted | ^41^ |
| T-47D | 48 hours | fixed 4% PFA, stained hematoxylin and eosin, counted | ^46^ |
| Hs578T | 48 hours | trypzinization and cell number counted | ^47^ |
| MCF10A | 48 hours | trypzinization and cell number counted | ^47^ |
| PC-3 | 0h, 6h, 24h, 48h | %cell migration = [1-(scratch area at Tx (Hrs)/scratch area at T0] | ^45^ |
| DU-145 | 0h, 24h, 48h, 72h | %cell migration = [1-(scratch area at Tx (Hrs)/scratch area at T0] | ^45^ |

Supplemental Table 6. Type of medium used in tissue culture insert invasion assays in lower chamber

| Cell line | Medium used with supplements in bottom chamber | Reference |
| --- | --- | --- |
| MCF7 | fibroblast conditioned medium, 50ug/ml ascorbic acid serum free DMEM (lower chamber) | ^50^ |
| MCF7 | lower chamber 500uL 10% FCS-DMEM | ^43^ |
| MDA-MB-231 | bottom chambers 750 ul serum free medium | ^46^ |
| MDA-MB-231 | RPMI 10% FBS | ^49^ |
| MDA-MB-231 | FBS and fibronectin in lower chamber | ^42^ |
| MDA-MB-231 | RPMI1640 20% FBS | ^48^ |
| MDA-MB-435 | fibroblast conditioned medium, 50ug/ml ascorbic acid serum free DME | ^50^ |
| MDA-MB-435 | RPMI1640 20% FBS | ^48^ |
| MDA-MB-453 | lower chamber 500uL 10% FCS-DMEM | ^43^ |
| SKBR3 | 500 uL RPMI 1640, 10% serum bottom | ^44^ |
| BT549 | FBS and fibronectin in lower chamber | ^42^ |
| Hs578T | complete medium with 10% FBS (750 µl) | ^47^ |
| MCF10A | complete medium with 10% FBS (750 µl) | ^47^ |
| T-47D | bottom chambers 750 µl serum free medium | ^46^ |
| PC-3 | 0.8ml serum free medium with 25ug/ml fibronectin | ^45^ |
| DU-145 | 0.8ml serum free medium with 25ug/ml fibronectin | ^45^ |

Supplemental Figure 1


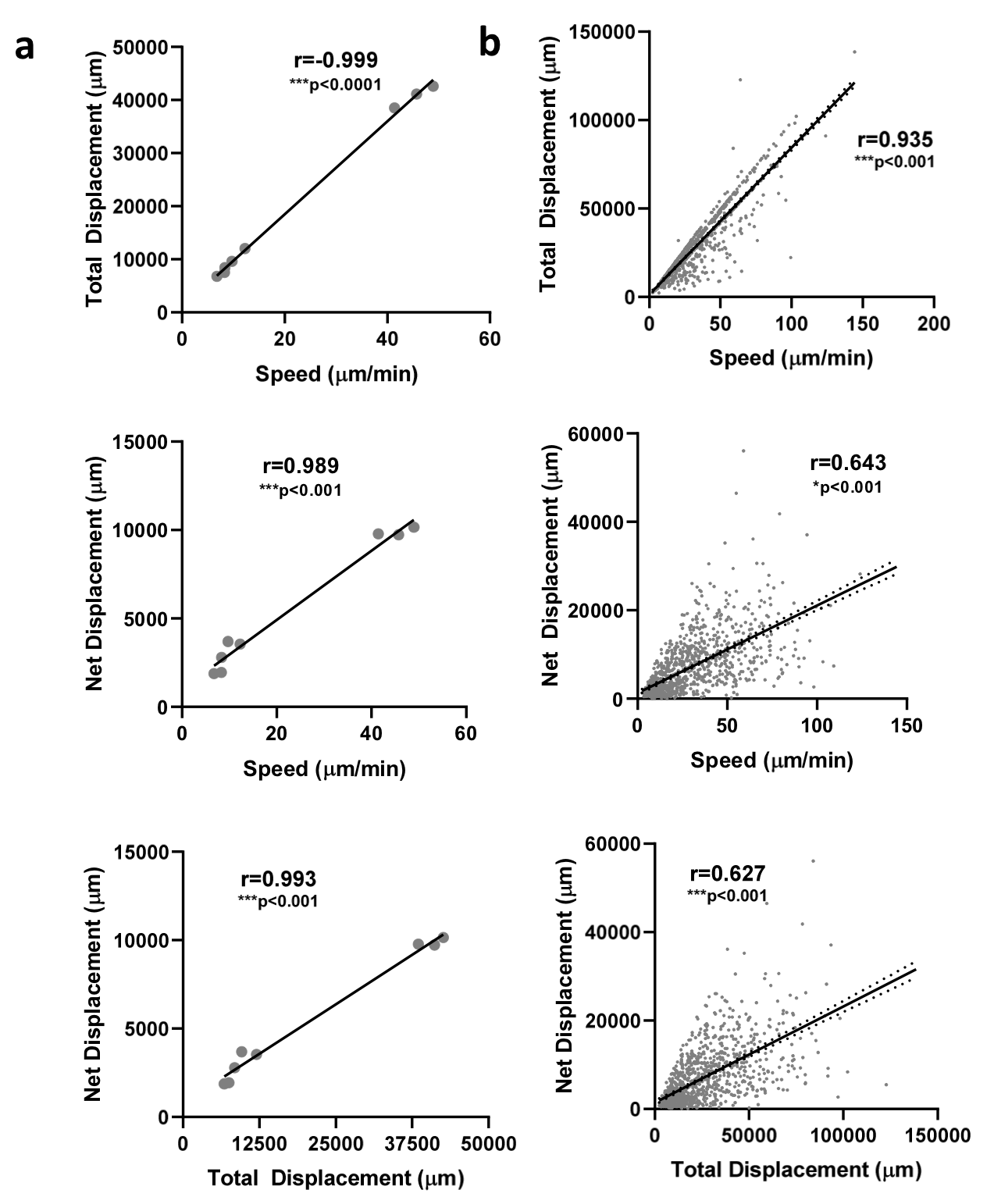


Figure S1: Correlation of metrics related to cellular displacement: A) Comparing total displacement, net displacement, and speed for the following four cell lines in 2D and 3D environments: G2, G34, G62, G528. B) Single cell data comparing the metrics mentioned previously for all cell lines and dimensions.

Supplemental Figure 2


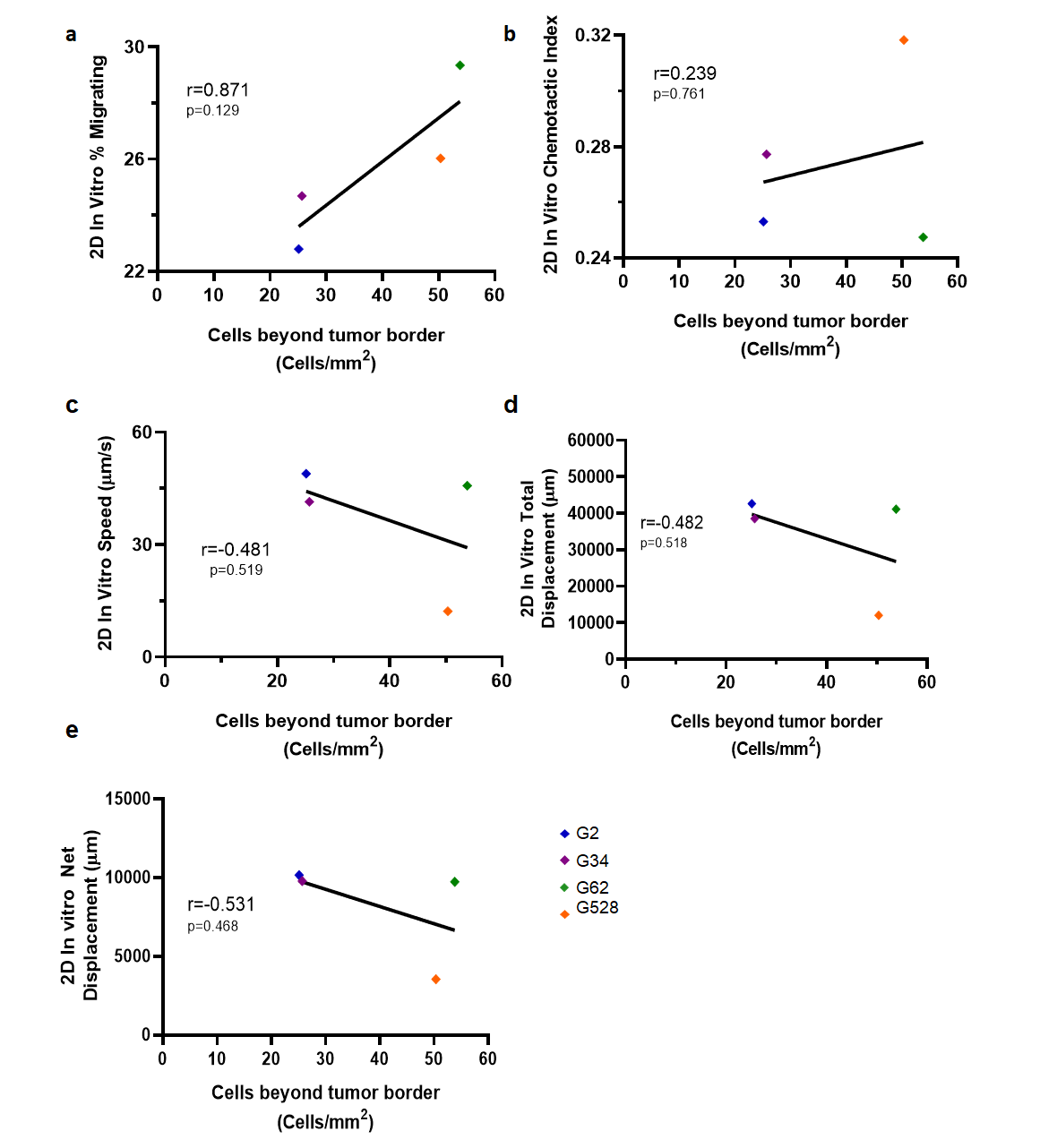


Figure S2: Motility metrics for glioma cells in 2D in vitro compared to in vivo invasion. a) Percent of cells migrating, b) chemotactic index, c) speed, d) total displacement, and e) net displacement graphed by glioma cell line vs. the number of invaded cells beyond the tumor border in vivo per mm2 of tissue. Pearson r correlation with p values listed on each graph.

Supplemental Figure 3


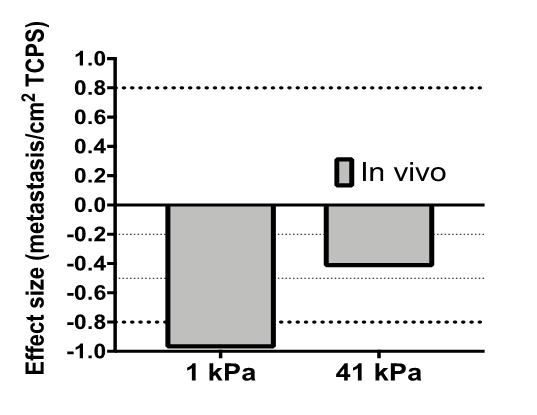


**Figure S3. Invasion of MDA-MB-231 cell line correlates with metastasis.** *In vivo* effect sizes compared to tissue culture polystyrene (TCPS) for cells cultured in soft (1kPa) or stiff (41kPa) hydrogels prior to implantation.

**Supplemental Figure 4**

**
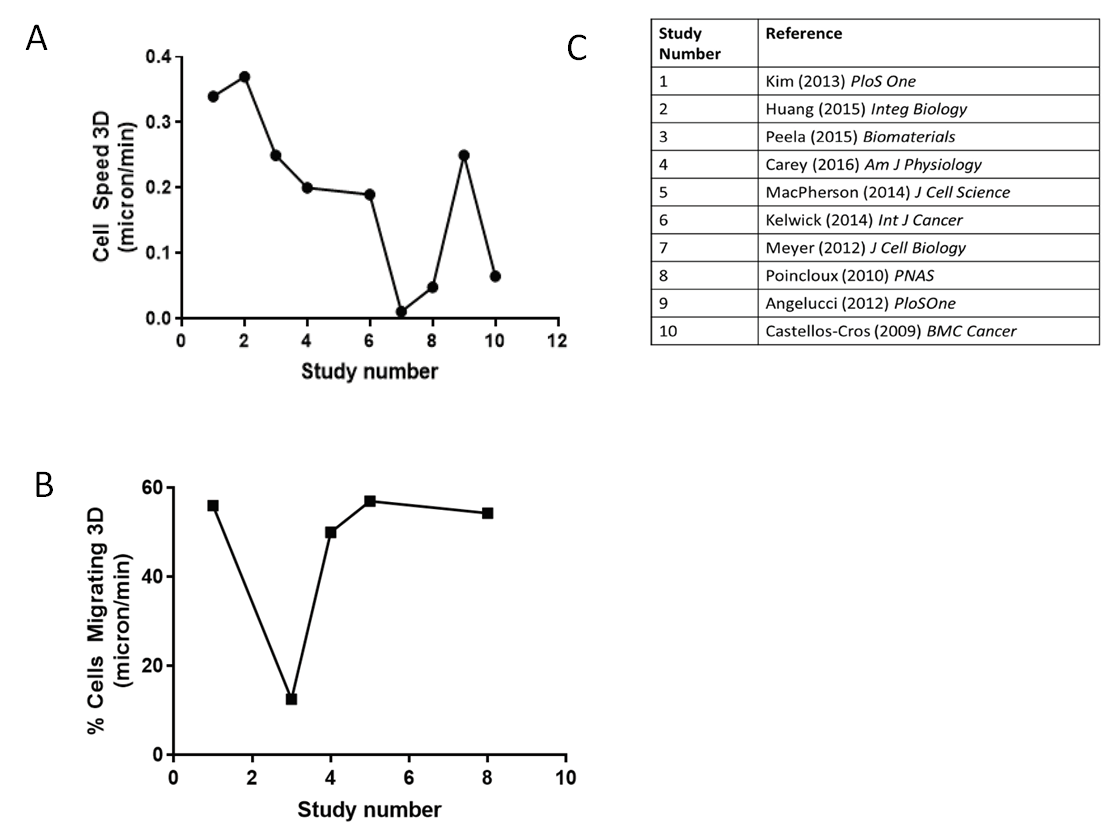
**

**Figure S4: Motility metrics for MDAMB231 cultured in Collagen I matrices and live imaged** A) Cell speed measured in 3D across studies B) % of cells migrating in 3D by study C) Table of studies from which data was extracted.
